# Decoding glioblastoma evolution and heterogeneity through mechanistic modeling: implications for clinical translation

**DOI:** 10.1186/s13046-026-03767-7

**Published:** 2026-06-29

**Authors:** Haowu Jiang, Wan Zhao, Hui Zhou, Rui Sun

**Affiliations:** 1 Department of Otolaryngology-Head and Neck Surgery, The First Affiliated Hospital of USTC, Center for Advanced Interdisciplinary Science and Biomedicine of IHM, Hefei National Research Center for Physical Sciences at the Microscale, Division of Life Sciences and Medicine, University of Science and Technology of China, No.96, JinZhai Road, Baohe District, Hefei, Anhui 230026 People’s Republic of China; 2 Department of Otolaryngology-Head and Neck Surgery, The First Affiliated Hospital of USTC, Division of Life Sciences and Medicine, University of Science and Technology of China, Hefei, 230001 People’s Republic of China; 3 Department of Respiratory Medicine, The First Affiliated Hospital of USTC, Division of Life Sciences and Medicine, University of Science and Technology of China (Anhui Provincial Cancer Hospital), Hefei, 230031 People’s Republic of China; 4 Department of Immunology, School of Basic Medical Sciences, Anhui Medical University, No.81, Meishan Road, Shushan District, Hefei, Anhui 230032 People’s Republic of China

**Keywords:** Glioblastoma, Tumor evolution, Heterogeneity, Stem cells, Brain organoids, 3D bioprinting, Organotypic brain slices, in vivo models, Artificial intelligence

## Abstract

Glioblastoma (GBM) is one of the most aggressive and lethal primary brain tumors in adults, characterized by dynamic clonal evolution and extensive genomic, cellular, spatial, and microenvironmental heterogeneity. Multi-omics studies have revealed that GBM follows complex evolutionary trajectories involving genetic, epigenetic, transcriptional, and immune-microenvironmental remodeling as tumors grow, adapt to the brain microenvironment, and acquire therapeutic resistance. Increasing evidence suggests that GBM may originate from aberrant neural stem or progenitor cells, including those residing in the subventricular zone, and that glioblastoma stem cells (GSCs) contribute to tumor propagation, heterogeneity, and recurrence. A key conceptual challenge is to reconcile hierarchical cancer stem cell models, in which GSCs are viewed as relatively stable tumor-propagating subpopulations, with dynamic state plasticity models, in which stem-like properties can be reversibly acquired or lost during transitions among proneural-like, mesenchymal-like, invasive, and therapy-tolerant states. Recent advances in single-cell profiling, spatial transcriptomics, lineage tracing, organoid culture, 3D bioprinting, genetically engineered models, and artificial intelligence (AI)-assisted computational modeling have substantially improved the ability to study these processes. However, no currently available model fully recapitulates human GBM heterogeneity, recurrence, treatment history, and tumor–microenvironment interactions. Therefore, model selection should be guided by clearly defined mechanistic questions rather than by reliance on any single platform. This review summarizes current advances in in vitro, ex vivo, in vivo, and computational models for studying GBM evolution and heterogeneity, and discusses how integrated model pipelines may improve preclinical drug testing, treatment-response prediction, and precision neuro-oncology.

## Introduction

Glioblastoma (GBM), classified as a World Health Organization (WHO) grade IV glioma, is the most common and lethal primary tumor of the central nervous system. The median overall survival of patients with newly diagnosed GBM is less than 15 months [1]. Despite the current standard of care, which includes maximal surgical resection followed by radiotherapy and temozolomide chemotherapy, recurrence is almost inevitable and is typically associated with rapid disease progression and poor clinical outcomes [1, 2]. Advances in genomic and transcriptomic sequencing technologies have substantially enhanced our understanding of the spatial and temporal heterogeneity of GBM, facilitating the identification of novel therapeutic targets and the development of more effective treatment strategies [3–8]. GBM exhibits extensive spatial intratumoral heterogeneity driven by branched evolutionary trajectories, with multiple molecular subtypes coexisting within individual tumors [3]. Consistent with this, three tumor-intrinsic transcriptional subtypes of *IDH*-wildtype GBM, namely proneural, mesenchymal, and classical, have been defined, with subtype multiplicity associated with increased intratumoral heterogeneity and microenvironmental complexity [4]. At the single-cell level, GBM displays substantial heterogeneity, characterized by diverse transcriptional programs related to oncogenic signaling, proliferation, immune response, and hypoxia that are variably expressed across individual cells [5]. Furthermore, pronounced spatial heterogeneity exists between tumor core and peripheral regions, where infiltrating neoplastic cells share a conserved gene expression signature across patients, suggesting a common mechanism of invasion [6]. Intertumoral heterogeneity in GBM at the genetic and cellular levels is also widely observed and is reflected in variable sensitivity to anticancer therapies [7]. In addition, recurrent GBM exhibits heterogeneity driven by phenotypic plasticity rather than genetic selection compared with primary tumors, with a greater proportion of tumors adopting a mesenchymal state under therapeutic pressure [8]. Collectively, intra- and intertumoral heterogeneity, which is closely associated with prognosis and therapeutic resistance, poses major challenges for the development of broadly effective therapies while creating opportunities for patient-specific treatment approaches.

The high malignancy of GBM, relative to other tumor types, is likely associated with its pronounced evolutionary capacity and intrinsic stem-like properties. The existence of a small, highly malignant subpopulation of cells in GBM has been confirmed by studies using transgenic animal models and human specimens [9–11]. These cells, referred to as glioblastoma stem cells (GSCs) or brain tumor-initiating cells (TICs), exhibit key stem cell properties such as self-renewal and multipotency, and, most importantly, demonstrate strong tumorigenic potential in vivo [10, 12]. In GBM research, the terms GSCs and TICs are often used interchangeably; however, they differ in definition and emphasis. GSCs typically refer to stem-like tumor cells within gliomas that possess self-renewal and differentiation capacity, whereas TICs are functionally defined by their ability to initiate tumor formation upon transplantation into animal models, regardless of stemness characteristics [13, 14]. Accordingly, GSCs are generally considered tumor-initiating, while TICs may encompass a broader and heterogeneous population, including GSCs as a subset. The prevailing view over recent decades is that GSCs originate from neural stem cells (NSCs) or multipotent or bipotent progenitor cells in the adult brain and play a central role in GBM initiation [10, 15, 16]. However, evidence also suggests that fully differentiated cells can undergo dedifferentiation and contribute to GBM formation [17]. The hierarchical cancer stem cell model posits that GBM is sustained by a small population of GSCs that drive clonal evolution and give rise to heterogeneous progenitor and differentiated tumor cell populations [15, 16]. Consistent with this model, studies have demonstrated that GBM is organized along a conserved neural tri-lineage hierarchy centered on glial progenitor-like cells, which represent a key cancer stem cell compartment and serve as the source of diverse tumor cell states [16, 18]. The key characteristics of GBM, including its evolutionary nature, intercellular heterogeneity, and treatment resistance, are likely associated with stemness features present in specific tumor cell subpopulations. An important question in GSC biology is how these cells maintain stem cell properties and initiate tumor formation across diverse brain microenvironments, given that normal NSCs rely on specialized neurogenic niches within specific brain regions to sustain self-renewal and differentiation into mature neural cell types. One possible explanation is that oncogenic mutations in neural stem or progenitor cells drive aberrant proliferation, enabling these cells to become less dependent on extrinsic regulatory cues while retaining high evolutionary potential and partial differentiation capacity. The subventricular zone is known to generate neurons and glial cells throughout life and serves as a source of stem cells and progenitors in the adult brain [19]. Other regions, such as the subgranular zone (SGZ) of the dentate gyrus, also contribute to adult neurogenesis [20]. Specific genetic alterations in neural stem or progenitor cells within these areas have been shown to drive gliomagenesis, and GSCs isolated from tumors often exhibit accumulated driver mutations, such as *EGFR* amplification and *TERT* promoter mutations [21, 22].

Malignant cells in GBM have been classified into four major cellular states: neural progenitor-like (NPC-like), oligodendrocyte progenitor-like (OPC-like), astrocyte-like (AC-like), and mesenchymal-like (MES-like) states [23]. Each of these cellular states includes cells with proliferative capacity, and their relative abundance is associated with specific genetic alterations, including mutations in *CDK4*, *PDGFRA*, *EGFR*, and *NF1* [23]. Thus, primary oncogenic mutations may arise from distinct neurodevelopmental lineage origins, contributing to divergent evolutionary trajectories and driving both intra- and intertumoral heterogeneity in GBM. Furthermore, these cellular states are influenced by microenvironmental cues and can undergo dynamic transitions, supporting the emerging dynamic state plasticity model of cancer, in which GBM identity is better understood as a heterogeneous and plastic mixture of cellular states rather than as a single fixed molecular subtype [17, 23]. This non-hierarchical model emphasizes that GSCs in GBM may not represent a permanently distinct subpopulation, but rather a reversible cellular state [17, 24, 25]. Accordingly, GBM cells can transiently acquire stem-like properties and subsequently transition toward more differentiated, mesenchymal, therapy-tolerant, or invasive phenotypes; conversely, more differentiated cells may reversibly reacquire stem-like states under the influence of intrinsic regulatory programs, microenvironmental signals, and therapeutic pressure [17, 24, 25].

The plasticity of GBM cells with stem-like features further contributes to tumor heterogeneity and phenotypic variability and is closely associated with therapeutic failure. Anticancer therapies, including temozolomide, can drive the interconversion of GBM stem-like cells and promote adaptive evolution, ultimately leading to treatment resistance [17, 26]. Phenotypic transitions among stem-like cell states have been shown to play a major role in mediating resistance to conventional therapies and other treatment strategies targeting tumor invasion, angiogenesis, and recurrence [24]. GBM cells can undergo proneural-to-mesenchymal transition (PMT) in response to therapeutic pressures, such as radiation, thereby contributing to tumor progression, treatment resistance, and recurrence [27, 28]. Recurrent GBM cells that are tolerant to therapy often display a shift toward a mesenchymal-like state [8]. Both genetic and epigenetic mechanisms regulate GSC plasticity and evolution. Multiple signaling pathways and epigenetic programs involved in phenotypic adaptation have been identified, including TNF-α/NF-κB signaling, ILK/STAT3 signaling, Notch signaling, and histone demethylation [28–30].

Recent multidimensional and large-scale analyses of human GBM specimens, integrating genomic, epigenomic, metabolomic, proteomic, and lipidomic profiling, have substantially expanded our understanding of the complex tumor ecosystem [31–33]. These studies have identified several key regulatory signaling pathways involved in tumor evolution and recurrence, including the PTPN11 signaling pathway and the HIF1A–FOSL2 axis [32, 33]. Notably, many of these pathways mediate the interplay between tumor cells and immune cells [33, 34]. GBM remains a therapeutically challenging malignancy, partly due to its complex interactions with the immune system. The tumor microenvironment (TME) of GBM is characterized by abundant infiltration of immunosuppressive myeloid cells and a limited number of functionally exhausted T cells [35–37]. Tumor-associated macrophages (TAMs), the predominant myeloid population, exhibit marked phenotypic heterogeneity shaped by local microenvironmental cues [38, 39]. These include GBM-associated microglia and distinct macrophage subsets with lipid metabolism–associated, hypoxia-induced, and interferon-responsive profiles. Functional alterations in these populations are closely linked to tumor growth dynamics [34, 40]. The interaction between GBM cells and immunosuppressive TAMs drives the evolution of distinct tumor subclones with unique genetic and epigenetic signatures [4]. Spatiotemporal changes in TAM function, occurring in parallel with TME remodeling, establish positive feedback loops that promote GBM survival and expansion [38, 41, 42].

Overall, the incurability of GBM is attributable to multiple factors involving both tumor cell–intrinsic and microenvironmental features. GBM exhibits extensive spatial intratumoral heterogeneity at both molecular and cellular levels, as well as marked intertumoral heterogeneity, reflected in differences between primary and recurrent tumors and between treatment-naïve and treated samples [43, 44]. This heterogeneity is partly driven by diverse populations of brain tumor-initiating cells (TICs) and their progeny, arising from distinct genetic and epigenetic alterations [45]. GBM also displays pronounced intrinsic plasticity and epigenetically driven phenotypic transitions, which enhance resistance to multiple antitumor therapies and contribute to rapid recurrence [4, 40, 46]. The dynamic presence of GSC states within GBM subpopulations further promotes tumor survival and invasiveness. In addition, tumor–immune interactions, including increased TAM infiltration and paracrine signaling networks, are key contributors to recurrence and provide a framework for identifying tumor-intrinsic and microenvironmental therapeutic targets.

Despite substantial progress in elucidating the mechanisms underlying GBM pathogenesis and progression, effective and broadly applicable therapeutic strategies remain limited [47, 48]. One major obstacle is the inadequacy of current preclinical GBM models. Mechanistic GBM models, including both experimental and computational systems, are intended to recapitulate the cellular, molecular, genetic, and microenvironmental processes that drive tumor evolution, intratumoral heterogeneity, invasion, and treatment resistance. However, modeling GBM remains challenging because of its extensive spatial and temporal heterogeneity, diffuse brain infiltration, and dynamic interactions with neural, vascular, immune, and stromal components of the brain microenvironment. Although existing models reproduce some key features of GBM biology, they often fail to capture the dynamic interactions between GBM cells and their microenvironment during tumor progression and therapeutic response. Moreover, interpatient variability in the initiating oncogenic events and genetic alteration patterns that drive GBM formation makes it difficult to rely on a single standardized model for drug screening and therapeutic evaluation [7, 49]. Newly diagnosed and recurrent GBMs also exhibit substantial genetic, epigenetic, and cellular divergence, which is closely associated with variable therapeutic responses and patient outcomes [44, 50].

**Fig. 1:**
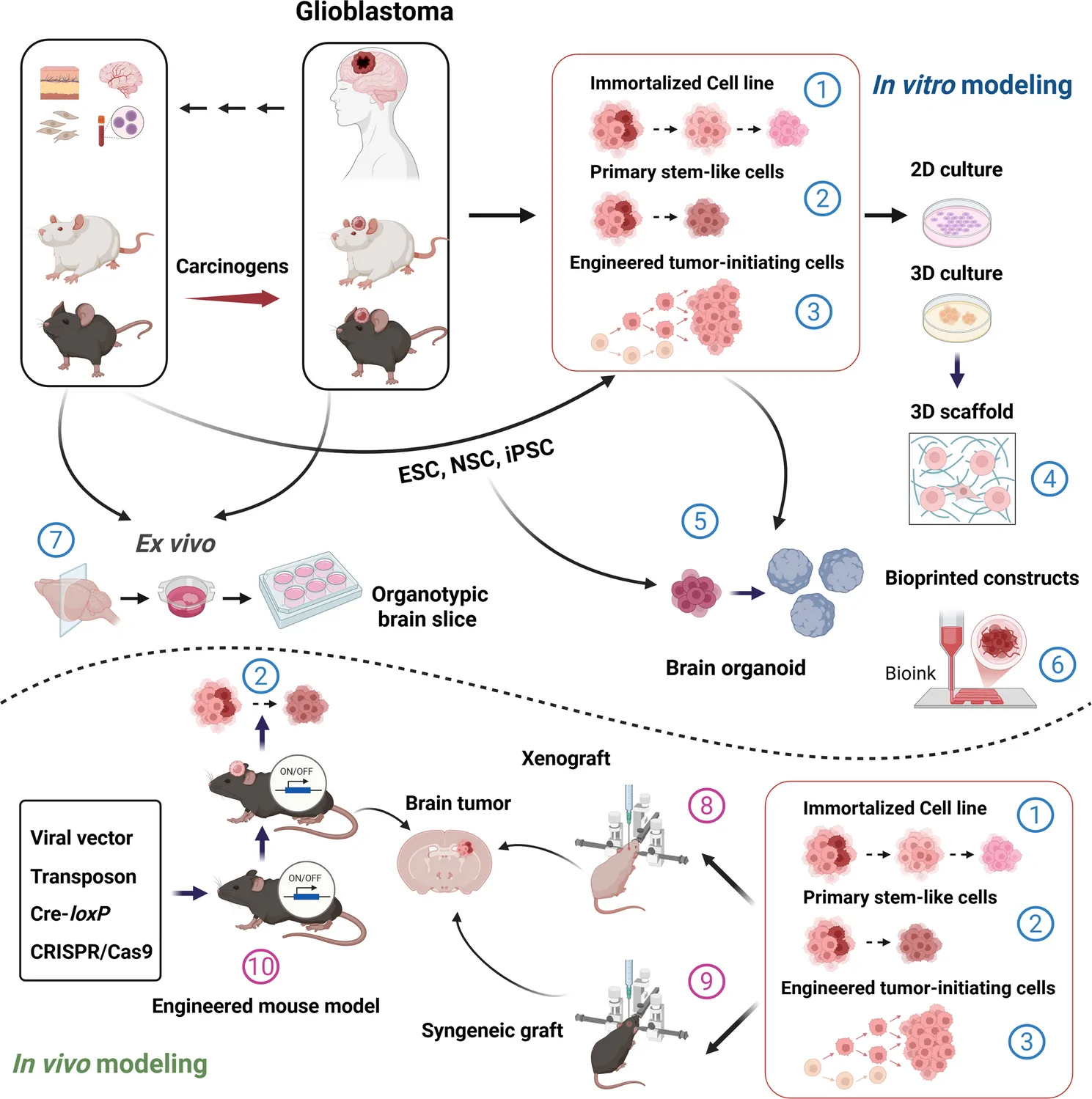
Preclinical models for GBM research across in vitro, ex vivo, and in vivo platforms. The principal strategies used to model GBM using in vitro, ex vivo, and in vivo systems. Tumor sources include immortalized cell lines (1), primary stem-like cells (2), and engineered tumor-initiating cells (3). Immortalized GBM cell lines are established from surgically resected human tumors or generated in mice via carcinogen exposure and serial passaging. Primary stem-like cells, or glioblastoma stem-like cells (GSCs), are isolated directly from fresh patient tumor tissue. Engineered tumor-initiating cells are generated by introducing genetic alterations into pluripotent stem cells, such as embryonic stem cells (ESCs), neural stem cells (NSCs), or induced pluripotent stem cells (iPSCs). These tumor sources are applied in two-dimensional (2D) and three-dimensional (3D) culture systems, including scaffold-based models (4), brain organoids (5), and bioprinted constructs using bioinks (6), to recapitulate tumor behavior and the tumor microenvironment (TME) in vitro. Organotypic brain slice cultures (7) provide an ex vivo platform that preserves native brain architecture for functional and therapeutic studies. In vivo models include xenografts (8), where human tumor cells are implanted into immunodeficient mice, and syngeneic grafts (9), in which murine GBM cells are implanted into immunocompetent mice. Genetically engineered mouse models (GEMMs) (10) are established using gene-editing technologies such as viral vectors, transposons, Cre-*loxP* recombination, or CRISPR/Cas9, enabling the study of spontaneous tumor initiation and progression

**Fig. 2:**
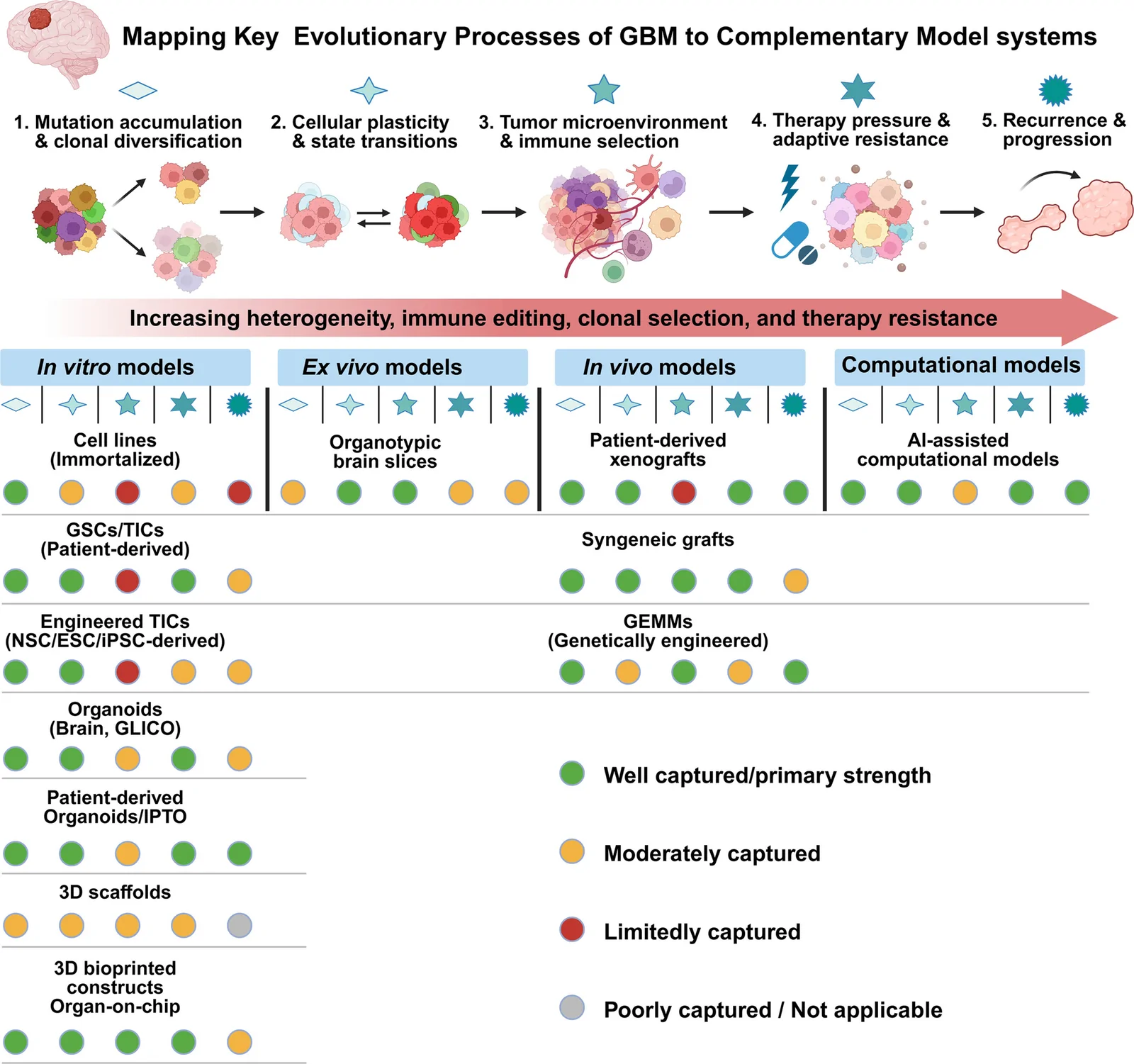
An integrative framework for modeling GBM evolution. This schematic summarizes how major GBM evolutionary processes can be interrogated using complementary experimental and computational models. The upper panel illustrates a simplified evolutionary trajectory, including mutation accumulation and clonal diversification, cellular plasticity and state transitions, tumor microenvironment interactions and immune selection, therapy pressure and adaptive resistance, and recurrence or progression. The lower panel maps these processes to commonly used model systems, including in vitro models, ex vivo organotypic brain slices, in vivo xenograft, syngeneic and genetically engineered mouse models, and AI-assisted computational models. Colored circles indicate the relative capacity of each model to capture specific evolutionary features: green, well captured or primary strength; yellow, moderately captured; red, limitedly captured; and gray, poorly captured or not applicable. This framework highlights that no single model fully recapitulates all aspects of GBM evolution, and that integrated use of complementary models is required to study tumor heterogeneity, microenvironmental selection, treatment resistance, and recurrence. GBM, glioblastoma; GSCs, glioblastoma stem cells; TICs, tumor-initiating cells; NSCs, neural stem cells; ESCs, embryonic stem cells; iPSCs, induced pluripotent stem cells; 3D, three-dimensional; GLICO, glioma cerebral organoids; IPTO, individualized patient tumor organoid; GEMMs, genetically engineered mouse models; tumor microenvironment; AI, artificial intelligence

Over the past decade, a wide range of methodologies has been developed to investigate the mechanisms underlying GBM pathogenesis and to explore potential therapeutic strategies. However, the rapid accumulation of new insights into GBM biology has exposed limitations in existing preclinical models, as many therapies demonstrating efficacy in preclinical settings fail to translate into clinical benefit [47]. This discrepancy underscores the urgent need for more physiologically relevant and predictive model systems. Here, we outline the current spectrum of in vitro, ex vivo, and in vivo experimental models (Fig. 1), spanning cellular to organismal levels, that are widely used to study GBM progression, tumor evolution, heterogeneity, and therapeutic response. These models include established cell lines, primary tumor-derived cultures, engineered TICs, scaffold-based systems, brain organoids, bioprinted tissue constructs, organotypic brain slice cultures, orthotopic transplantation models, genetically engineered *de novo* models, and artificial intelligence (AI)-assisted computational models. Each of these methodologies contributes to understanding key characteristics of GBM, including mutation accumulation, cellular plasticity, immune selection, and therapy resistance (Fig. 2). We further discuss the strengths and limitations of each system, as well as the specific biological mechanisms they are best suited to interrogate, to guide the selection of appropriate models for defined research objectives (Table 1). To facilitate effective translation from preclinical research to clinical application, the integrated use of complementary GBM models is recommended for evaluating the efficacy and underlying mechanisms of novel therapies. Future advances in personalized and physiologically relevant model platforms are expected to refine our understanding of GBM pathogenesis and accelerate the development of innovative therapeutic strategies.

**Table 1:** Comparative overview of major GBM model systems for studying evolution, heterogeneity, and therapeutic response

Model system	Strengths	Limitations	Evolutionary features captured	Translational relevance	Representative references
Immortalized GBM cell lines	Reproducible, scalable, low cost, genetically tractable, suitable for rapid drug screening	Long-term culture adaptation, reduced heterogeneity, limited TME representation, risk of misidentification	Tumor cell-intrinsic growth, invasion, pathway perturbation, drug response	Useful for preliminary mechanistic studies and high-throughput screening, but limited predictive value	[54–70]
Primary tumor-derived GSCs or TICs	Preserve patient-specific mutations, stem-like properties, tumor-propagating capacity, and therapeutic vulnerabilities	Loss of TME components, culture-driven selection, passage-dependent drift, variable reproducibility	Stemness, clonal propagation, plasticity, therapy-induced adaptation, primary-to-recurrent progression	Valuable for individualized drug testing and orthotopic xenograft generation	[71–84]
Engineered TICs from NSCs, ESCs, or iPSCs	Genetically defined, experimentally controllable, useful for testing mutation order and driver cooperation	Artificial transformation, incomplete patient heterogeneity, iPSC line variability, off-target editing effects	Tumor initiation, cooperative oncogenesis, cell-of-origin effects, lineage restriction, early evolution	Useful for causal mechanistic studies and genotype-specific therapeutic testing	[85–95]
Scaffold and hydrogel-based 3D models	Tunable matrix stiffness, composition, porosity, degradability, and ligand density	Limited cellular complexity, incomplete immune and vascular components, material-dependent variability	Mechanotransduction, matrix-dependent invasion, mesenchymal-like transition, matrix-mediated drug resistance	Useful for testing invasion mechanisms, drug delivery, and matrix-targeted therapies	[96–102]
Brain organoid and GLICO models	Provide human brain-like neural context; enable modeling of tumor invasion and tumor–neural interactions	Developmental rather than adult-like tissue, limited vascularization and immune infiltration, protocol variability	Tumor initiation, GSC invasion, tumor microtube formation, tumor–brain interaction, host-tissue remodeling	Useful for modeling tumor–brain interactions and testing anti-invasive therapies	[52, 103–117]
Patient-derived GBM organoids	Preserve parental tumor histology, cellular diversity, gene-expression programs, and mutational profiles	Limited long-term immune, vascular, and neural representation; batch variability; moderate scalability	Intratumoral heterogeneity, tumor architecture, treatment response, therapy-resistant states	Strong potential for patient-specific drug testing, biobanking, and functional precision oncology	[105, 106, 118–121]
IPTO and chimeric organoid models	Combine patient tumor explants with brain-like host tissue; retain tumor-intrinsic and brain microenvironmental cues	Technically complex, limited standardization, cost and scalability challenges	Invasion, cellular plasticity, clonal selection, tumor–brain interactions, treatment response	Promising patient-specific platform for functional drug testing and precision oncology	[122]
3D bioprinted constructs and GBM-on-chip models	Spatially controlled multicellular architecture; can incorporate macrophages, astrocytes, endothelial cells, pericytes, ECM, perfusion, and gradients	Bioink limitations, technical complexity, variable vascular maturity, standardization challenges	TME-driven heterogeneity, immune modulation, vascular niche effects, hypoxia, drug diffusion, treatment resistance	Useful for drug delivery studies, vascularized modeling, high-content testing, and personalized therapy screening	[53, 123–142]
Organotypic brain slice cultures	Preserve native brain architecture, regional cues, local cellular diversity, and microenvironmental interactions	Short-term viability, dependence on fresh tissue, protocol variability, limited systemic immunity	Local invasion, perivascular migration, microenvironmental interaction, ex vivo treatment response	Valuable for patient-relevant invasion assays and short-term therapeutic sensitivity testing	[143–154]
Patient-derived xenografts and orthotopic xenografts	Preserve invasive growth and patient-derived tumor features in vivo; enable survival and therapeutic studies	Usually require immunodeficient hosts, limited immune modeling, engraftment variability, time and cost	Tumor growth, invasion, clonal selection, therapy response, recurrence-like progression	Useful for preclinical efficacy testing, but limited for immunotherapy unless humanized systems are used	[159–165]
Syngeneic mouse models	Immune-competent host, reproducible implantation, suitable for immunotherapy studies	Murine tumor genetics may not fully match human GBM; some models show high immunogenicity	Tumor–immune interactions, immune selection, macrophage and T-cell modulation, therapy response	Valuable for immunotherapy, immune modulation, and combination therapy testing	[37, 62–64, 169–194]
Genetically engineered mouse models	Tumors arise de novo in an immune-competent brain; defined genetic drivers; useful for tumor initiation studies	Long generation time, lower throughput, species differences, limited interpatient heterogeneity	Tumor initiation, mutation-driven evolution, cell-of-origin effects, immune co-evolution, progression	Strong mechanistic relevance for gliomagenesis and driver validation	[161, 195–212]
AI-assisted computational models	Integrate multi-omics, spatial, imaging, histopathological, and clinical data; scalable analytical power	Dependent on data quality, cohort size, interpretability, validation, and regulatory acceptance	Evolutionary trajectory inference, cell-state transition prediction, treatment-response modeling, risk stratification	Promising for patient stratification, model selection, therapeutic prediction, and precision neuro-oncology	[5, 8, 23, 213–218]

This table summarizes the principal in vitro, ex vivo, in vivo, and computational model systems used in GBM research, highlighting their major strengths, limitations, evolutionary features captured, translational relevance, and representative references. The comparison is intended to guide model selection according to specific mechanistic questions, including tumor initiation, clonal evolution, cellular plasticity, tumor–microenvironment interactions, immune selection, invasion, therapy resistance, and treatment-response prediction. Because no single model fully recapitulates the biological complexity of human GBM, complementary use of multiple model systems is often required to improve mechanistic interpretation and clinical translation

*GBM* Glioblastoma, *GSCs* Glioblastoma stem cells, *TICs* Tumor-initiating cells, *NSCs* Neural stem cells, *ESCs* Embryonic stem cells, *iPSCs* induced pluripotent stem cells, *3D*, three-dimensional, *GLICO* Glioma cerebral organoids, *IPTO* Individualized patient tumor organoid, *TME* Tumor microenvironment, *AI* Artificial intelligence, *ECM* Extracellular matrix

## In vitro modeling of GBM

In vitro models offer a cost-effective and reproducible approach for investigating tumor cell-intrinsic properties in GBM research [51]. Cell cultures have been widely utilized, even though they are limited in their ability to recapitulate the dynamic evolution and heterogeneity of the GBM microenvironment. Nonetheless, in vitro cell culture systems remain highly amenable to chemical, biological, and genetic manipulation, making them widely used tools for basic mechanistic studies. Now, they also serve as essential platforms for target identification, drug sensitivity testing, and high-throughput screening and analysis. Most in vitro models function as foundational tools that are linked to the establishment of in vivo systems. Additionally, in vitro studies provide complementary insights that extend findings obtained from in vivo models. So far, in vitro cell culture systems can be broadly classified into two categories based on their cellular organization: two-dimensional (2D) and three-dimensional (3D) cultures. Among in vitro models, 3D culture systems that incorporate interactions between GBM cells and other non-tumor components have advanced rapidly, demonstrating strengths in investigating tumor invasion, evolutionary dynamics, and microenvironmental remodeling [52, 53]. These innovative systems may improve the efficiency of new therapeutic testing.

### Cell lines

Immortalized malignant glioma cell lines from different species were developed early and are typically cultured in 2D systems. These include human cell lines such as U87MG, U118MG, U251, T98G, LN-18, and LN-229 [54, 55]; rat cell lines such as C6, 9 L, and F98 [56]; and mouse cell lines such as GL261 and G422 [57, 58]. These cultures are generally maintained in serum-containing media and some of them can also be propagated as spheres in serum-free conditions. Most human glioma cell lines are derived from resected tumor tissues of patients with high-grade gliomas and can be maintained through successive passaging in vitro [59, 60]. Nearly all rat glioma cell lines have been isolated and purified from chemically induced gliomas in rats, typically generated using carcinogenic agents such as nitrosomethylurea (NMU) [56]. The majority of mouse glioma cell lines, including GL261, G422, and CT-2 A, were derived from malignant gliomas induced by intracerebral implantation of chemical carcinogens such as methylcholanthrene [61]. SB28, a mouse GBM cell line established in 2012 from gliomas induced by *Sleeping Beauty* (SB) transposon-mediated genetic modifications targeting *Pdgfb*, *Trp53* and *Nras*, recapitulates several features of human GBM [37, 62–64]. These glioma cell lines have undergone selection through repeated intracranial transplantation and serial passaging over several generations. Thus, immortalized cell lines used in GBM study exhibit several characteristics similar to those of cancer stem cells, including high proliferative capacity following multiple rounds of clonal selection. It is also evident that these cell cultures lose the cellular and genetic heterogeneity inherent in their parental tumors. Furthermore, although they can be extensively expanded and are amenable to genetic and pharmacological manipulation without ethical constraints, they fail to reflect many tumor cell-extrinsic factors involved in GBM progression under in vitro culture conditions. Notably, many of these cell lines, including U251, F98, GL261, and SB28, are tumorigenic and capable of forming tumors in animals following subcutaneous or intracranial injections [56, 62, 65]. Therefore, they have been widely utilized for the establishment of in vivo models. It can be inferred that GBM cells with stem-like properties are able to produce highly heterogeneous tumors when supported by a conducive microenvironment.

These GBM cell lines harbor distinct genetic alterations and phenotypic features, making them commonly used tools for investigating genotype–phenotype relationships, candidate driver pathways, and therapeutic vulnerabilities [66]. Genetic and pharmacological profiling has been performed in these cells to evaluate alterations in pathways such as TP53, PTEN/PI3K, EGFR/RTK, RAS/MAPK, and cell-cycle regulation [67]. Because these cell lines can be expanded in vitro and are scalable, reproducible, and amenable to genetic perturbation approaches, they are widely used to study tumor growth, invasion, treatment response, and evolutionary adaptation under defined experimental conditions [68]. When used in vivo, human GBM cell lines are commonly implanted into immunodeficient mice to assess tumorigenicity and therapeutic response, whereas syngeneic murine glioma cell lines such as GL261, CT-2 A, and SB28 can be implanted into immunocompetent mice and are particularly useful for evaluating immune-dependent therapeutic strategies [37, 64, 69]. Nevertheless, these models should be interpreted with caution, as prolonged culture, selection pressure, and cell-line misidentification may alter their genetic and phenotypic properties [70].

### Primary tumor-derived cells

Primary cell cultures derived from GBM patients or tumor-bearing animals have garnered considerable attention, as they can partially preserve the heterogeneity, mutational landscape, and tumor-propagating properties of the original tumors. These primary GBM cells can be cultured either as 2D adherent monolayers or as 3D tumor spheroids in suspension, without requiring an attachment matrix. The culture medium for these cells resembles that used for NSCs, supplemented with epidermal growth factor (EGF) and fibroblast growth factor 2 (FGF-2), and devoid of serum to better maintain their stem-like properties [71]. Compared with serum-adapted glioma cell lines, these cells retain key phenotypic and genotypic features of parental tumors, supporting their value in mechanistic and translational modeling. Primary GBM cells, often termed GSCs or TICs, have been widely used to investigate tumor-propagating capacity, genetic and transcriptional programs, invasive behavior, therapeutic resistance, and patient-specific drug responses [72–74]. Large-scale patient-derived GSC resources have further demonstrated that these cultures can represent major GBM molecular subtypes and retain GBM-characteristic genomic lesions, making them useful platforms for modeling interpatient heterogeneity and linking molecular alterations to functional phenotypes [75].

GSC models are particularly useful for interrogating of stemness-associated signaling, clonal evolution, cellular plasticity, and therapy-induced adaptation under defined experimental conditions. They have been reported to express NSC markers, including Nestin, SOX2, and NANOG [76]. Signaling pathways such as Notch, PI3K, Wnt/β-catenin, TGF-β, and DNA damage response pathways have been implicated in maintaining GSC self-renewal, invasion, and resistance to radiotherapy and chemotherapy [77–79]. GSCs are also experimentally tractable for studying GBM evolution. Genetic modification of GSCs through retroviral, lentiviral, or CRISPR-based approaches enables functional interrogation of candidate drivers regulating tumor initiation, plasticity, invasion, and therapeutic resistance [16]. GSC models also facilitate individualized studies of GBM and enable investigation of the mechanisms underlying progression from primary to recurrent tumors. Primary GSCs with limited passage numbers retain tumorigenic potential and are frequently used to establish orthotopic xenograft models, which can partially recapitulate the histological, molecular, and invasive features of the original tumors. Clinically relevant applications include evaluating sensitivity to temozolomide, optimizing radiotherapy regimens, assessing targeted agents, and identifying patient-specific vulnerabilities. Moreover, intracerebral implantation of GSCs followed by sequential in vivo passaging provides a useful approach for studying tumor evolution, clonal selection, and intratumoral heterogeneity within the brain microenvironment. Notably, emerging single-cell and lineage-tracing studies suggest that stem-like phenotypes in GBM are not fixed properties of a distinct cellular subpopulation but instead arise from dynamic tumor cell plasticity shaped by microenvironmental cues [17]. These findings highlight the utility of GSC systems for modeling both hierarchical tumor propagation and dynamic cellular plasticity.

However, these models also have limitations. GSCs beyond 20 passages are generally not recommended for use, as prolonged culture may lead to genetic drift and metabolic alterations [80, 81]. In addition, GSCs may display distinct characteristics when cultured as 2D monolayers versus 3D aggregates [82]. GSCs grown as monolayers are experimentally convenient to handle but fail to reproduce their native growth patterns and behavioral characteristics observed in vivo [83]. In contrast, 3D cultures more closely mimic the in vivo growth state of GSCs. However, timely subculturing is required to prevent tumorsphere overgrowth and subsequent alterations in cellular states [84].

### Engineered tumor-initiating cells

Since GSCs exhibit many stem cell-like characteristics, a reductionist strategy for generating TICs in vitro involves retrofitting normal stem cells with oncogenic mutations using genetic engineering technologies. Primary NSCs, considered a reliable starting material for generating GBM cells, can be isolated directly from fetal or adult brain tissues of both human and animal origin. These cells can be cultured in suspension as neurospheres in vitro and retain a diploid karyotype along with multipotent differentiation capacity over multiple passages. In research, they are often cultured as laminin-adherent monolayers to facilitate experimental procedures such as clonal screening and expansion. Nevertheless, ethical concerns regarding the procurement and potential misuse of human NSCs have limited their large-scale and widespread application in GBM modeling [85]. In addition, data obtained from a limited number of human NSC-derived GBM cell lines are insufficient to capture the full extent of intertumoral heterogeneity. The use of NSCs derived from human embryonic stem cells (ESCs) in the development of in vitro GBM models presents similar ethical challenges.

Recent studies have highlighted the utility of human induced pluripotent stem cells (iPSCs) for generating brain-specific cell types and establishing models of malignant brain tumors [86–88]. Pluripotent stem cells can be generated by introducing a defined set of transcription factors, such as *Oct4*, *Sox2*, *c-Myc*, and *Klf4*, into somatic cells, including fibroblasts, skin cells, or blood cells [89, 90]. These iPSCs exhibit characteristics nearly identical to those of ESCs. Thus far, NSCs derived from iPSCs in vitro have provided an ethically acceptable alternative and enabled a continuous supply through scalable iPSC production. Genome-editing approaches, including lentiviral and retroviral transduction, transposon-mediated gene insertion, and CRISPR/Cas9-mediated mutagenesis, have been widely used to introduce oncogenic alterations into iPSC-derived NSCs [91, 92]. PTEN deficiency has been shown to reprogram human NSCs toward a GSC-like phenotype through PAX7 activation [93]. NSCs can be efficiently transformed into TICs by introducing CRISPR/Cas9-mediated mutations in *TP53* and *PTEN* in combination with lentiviral transduction-mediated overexpression of oncogenes such as EGFRvIII [94]. These models can therefore be used to investigate mutation order, cooperative oncogenic interactions, cell-of-origin effects, lineage-restricted differentiation, metabolic reprogramming, invasive behavior, and therapy-induced selection. Engineered TICs generated in vitro offer substantial advantages for studying clonal evolution and metabolic pathways that sustain tumor aggressiveness, as well as for personalized therapeutic screening in GBM treatment. These transformed cells have been shown to be tumorigenic, forming tumors following intracranial transplantation into immunocompromised xenogeneic or immunocompetent allogeneic hosts [95]. In vivo, iPSC-derived GBM models are commonly used to investigate genetically defined mechanisms of gliomagenesis and tumor evolution, partially recapitulating GBM heterogeneity and providing a useful platform for studying tumor–microenvironment interactions. However, meticulous experimental handling is essential to ensure data reliability and reproducibility. In addition, differentiation efficiency, clonal variability among iPSC lines, and off-target editing effects can affect reproducibility and the interpretation of biological findings.

### Scaffolds

Scaffold-based systems have been designed to investigate the interactions between GBM cells with the microenvironment [96, 97]. Scaffolds serve as artificial extracellular matrices to mimic the TME. Based on their rigidity, scaffolds are classified into two types: rigid scaffolds and hydrogels. Rigid scaffolds are typically composed of synthetic polymers such as polylactic acid (PLA), polyglycolic acid (PGA), and polycaprolactone (PCL), as well as natural substances like collagen. These materials provide a stable, porous structure that supports cell attachment, proliferation, and long-term culture. Rigid scaffolds are particularly useful for studying mechanotransduction, cell-matrix interactions, and tumor invasion within structurally defined environments [98]. Yet, a major limitation of these systems is their inability to replicate the dynamic and continuous remodeling of the TME that occurs during tumor growth and invasion, as the mechanical properties of most synthetic polymers lack sufficient tunability to accurately mimic these changes.

Hydrogels, on the other hand, are soft, water-swollen networks that consist of synthetic polymers, natural materials, and biomolecules, including polyethylene glycol (PEG), alginate, hyaluronic acid, fibrinogen, gelatin, and Matrigel [99]. They are hydrophilic and more closely resemble the texture of brain tissue, making them a major platform for modeling GBM behaviors such as cell proliferation, invasion, drug sensitivity, and cell–cell or cell–matrix interactions in vitro. Recently, hydrogel-based systems have advanced considerably, demonstrating substantial versatility and precise tunability in both composition and mechanical stiffness. Studies utilizing hydrogel-based models have identified several factors that influence GBM invasion, including the vascular niche and the viscoelastic properties of the extracellular matrix [100, 101]. Microenvironmental stiffness has been shown to promote mesenchymal-associated phenotypic transitions in GBM cells, including the induction of CTGF and YAP/TAZ signaling [102]. Overall, scaffold-based models, as a class of in vitro 3D culture systems, enable the investigation of the roles of extracellular matrix components and matrix-derived cues within the TME. They also show considerable potential for optimizing drug delivery strategies and predicting therapeutic responses in GBM research.

### Brain organoids

**Fig. 3:**
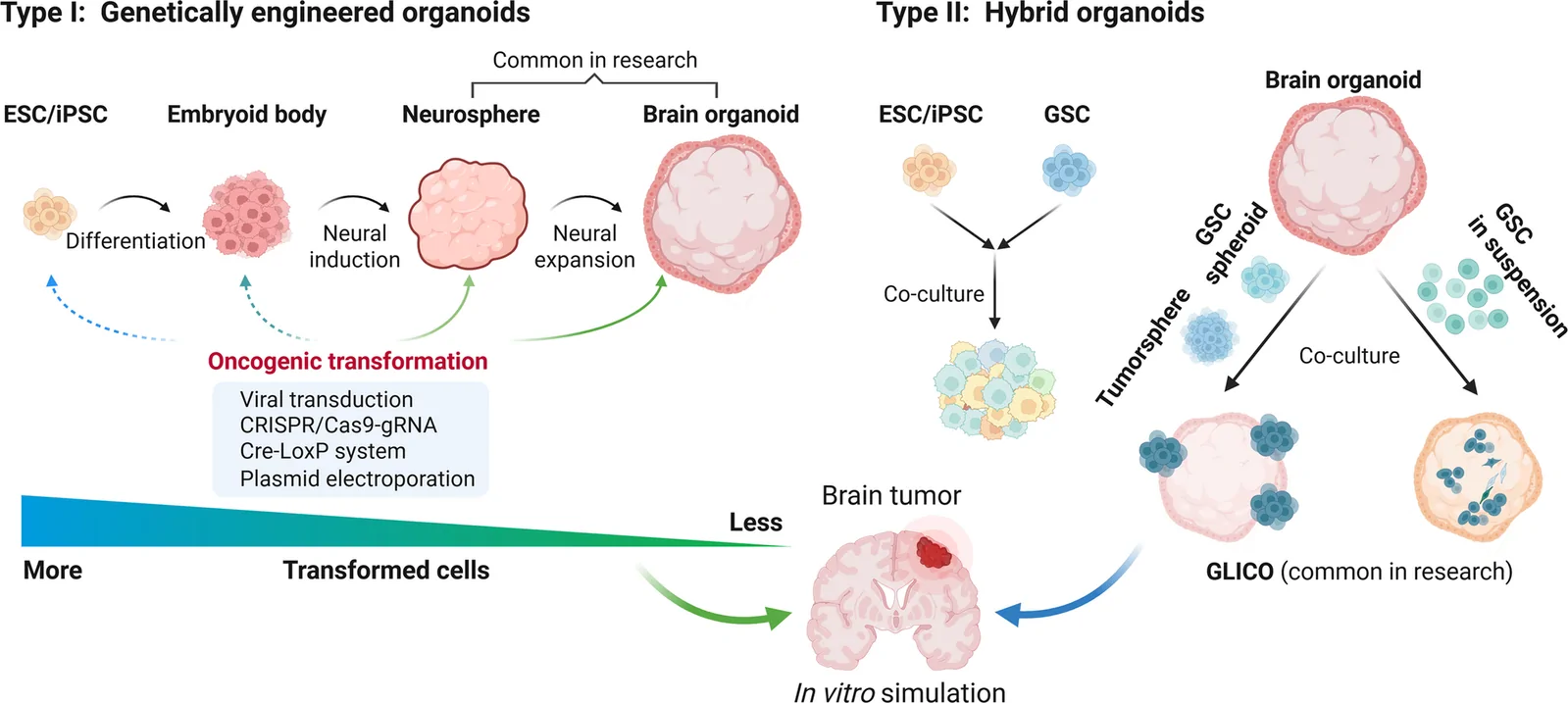
Overview of brain organoid-based models for GBM. Two major strategies for modeling GBM with brain organoids derived from pluripotent stem cells. Type I: Genetically engineered organoids. Human embryonic stem cells (ESCs) or induced pluripotent stem cells (iPSCs) undergo sequential differentiation to form embryoid bodies, followed by neural induction to generate neurospheres and subsequent expansion into brain organoids. During this developmental process, oncogenic transformation can be introduced at various stages using approaches such as viral transduction, CRISPR/Cas9-mediated gene editing, Cre-*loxP* recombination, or plasmid electroporation. The timing of genetic manipulation is critical, as early-stage transformation yields a higher proportion of transformed cells within the resulting organoids, whereas late-stage transformation is more commonly used to model GBM initiation and progression in a genetically controlled environment. Type II: Hybrid organoids. This strategy involves co-culturing patient-derived GSCs or tumorspheres with ESCs/iPSCs or their derived brain organoids. GSCs can be incorporated in two distinct ways to generate GLICO (glioblastoma cerebral organoid) models: (1) GSC spheres are directly co-cultured with brain organoids, or (2) dissociated GSCs are added as suspended cells to evaluate GBM invasion and interactions with host tissue. GLICO models are commonly used to investigate tumor–host interactions, GSC invasion dynamics, and therapeutic responses in a 3D, physiologically relevant environment

To more accurately mirror the TME by incorporating the roles of non-tumor cells, advanced in vitro models such as brain organoids have been developed (Fig. 3). Organoid models represent a type of 3D culture system typically derived from pluripotent stem cells, such as ESCs and iPSCs [103]. Yoshiki Sasai’s pioneering work demonstrated that pluripotent stem cells can intrinsically recapitulate key features of human brain development in *vitro*, laying the foundation for brain organoid technology [104]. Unlike tumor organoids, which are usually derived from resected GBM patients explants and cultured in vitro [105, 106], brain organoids are self-organizing, brain-like tissues differentiated from pluripotent stem cells [104]. They contain diverse brain cell types, including neural and glial cells, that resemble those found during brain development [107]. Although tumor organoids retain components of the original tumor mass, including heterogeneous GBM cells and blood vessels within the TME [105], they fail to replicate interactions between tumor cells and normal brain cells, as most of these models do not support the long-term survival of multiple brain cell types. While GSCs can be maintained in culture, other components of the TME are progressively lost over time. Brain organoids have been established as physiologically relevant models for studying human neurodevelopmental disorders and provide a platform for investigating brain tumor initiation, diffuse infiltration, cell–cell interactions, and therapeutic responses [52, 107].

Brain organoids, also termed cerebral or neural organoids, are created through a series of systematic procedures that may be adaptively modified across different laboratories worldwide. This process aims to reproduce the steps of neural tissue fate acquisition and morphogenesis in vivo [108]. It generally begins with the standard culture of pluripotent stem cells, such as ESCs or iPSCs, which give rise to embryoid bodies (EBs) under the influence of specific growth factors and nutrients [107–110]. These EBs are then directed through neural induction protocols, often involving the removal of pluripotency-maintaining signals and the addition of neural differentiation cues [107, 108]. Subsequently, the EBs are embedded in an extracellular matrix scaffold, such as Matrigel, to support 3D tissue organization [107, 108]. Further maturation, driven by agitation in spinning bioreactors or orbital shakers, enhances nutrient and oxygen exchange, fostering the self-organization of multiple brain regions and the formation of heterogeneous cytoarchitecture under defined differentiation conditions [107, 108]. Since 2008, multiple studies have reported the successful generation of brain organoids and their application in modeling human cortical development and neuronal biology [107, 109–111]. In 2018, two independent research groups were the first to utilize brain organoids to explore GBM biology [52, 112]. Both groups employed genome-editing techniques, including CRISPR/Cas9, to introduce oncogenic mutations into cerebral organoids, transforming them into malignant, tumor-like structures for studying GBM initiation and invasiveness. These transformed in vitro models also demonstrated potential for evaluating drug effects in brain tumors harboring specific combinations of oncogenic mutations. Shortly thereafter, additional studies emerged using stem cell-derived cerebral organoids as a platform for modeling GBM in vitro [113–116]. Thus far, two primary types of brain organoid models are widely utilized: (1) genetically engineered organoids, in which oncogenic mutations are introduced at various stages of pluripotent stem cell differentiation; and (2) patient-specific hybrid models, known as glioma cerebral organoids (GLICO), which are generated by co-culturing tumor-derived GSCs with either pluripotent stem cells or their derivative cerebral organoids [113, 114].

The first type of brain organoid model used in GBM research focuses on exploring the tumor’s intrinsic properties, including initiation, clonal evolution, expansion, and treatment resistance. This model has limitations in evaluating the authentic behavior of tumors in patients, as the TICs are artificially generated through the introduction of defined genetic alterations. The GLICO model has been refined compared with the first type by directly incorporating patient-derived GSCs to study tumor behaviors such as initiation and invasion [113]. In this system, human GBM spheroids spontaneously infiltrate early-stage cerebral organoids to generate hybrid organoid structures [117]. This model has been shown to more accurately phenocopy the histopathological and biological characteristics of patient GBM [113]. Further efforts have been made to improve the methodology of tumor invasion assays. Several parameters, such as the number of GSCs, the age of organoids, and the form of GSCs (single cells or spheres), have been optimized to better characterize GBM invasion [115]. Using the GLICO model, researchers have found that GSCs derived from recurrent GBM exhibit distinct invasive behavior compared to those from primary GBM [115]. An interconnected network of tumor microtubes facilitates the rapid growth and spread of GSCs within host organoids [113, 114]. In addition, these models can be used to elucidate communication between tumor cells and neurons, as growing evidence suggests that neuronal activity can influence glioma behavior. In summary, brain organoids represent a clinically relevant 3D in vitro platform for studying key features of GBM, including tumor invasion, evolution, and metabolic adaptation. These models have facilitated research in genetic analysis, personalized therapy, and drug development [114, 116]. However, several limitations remain, including: (1) culture protocols have not yet been standardized to fully support the differentiation and maturation of multiple brain cell types, such as oligodendrocytes and myelinated neurons; (2) the TME lacks infiltration by peripheral immune cells, including monocytes and neutrophils; (3) the vascular niche derived from endothelial cells remains underdeveloped; (4) the spatial architecture and heterogeneity of the TME may not be accurately recapitulated; and (5) because organoid generation is time-consuming and labor-intensive, large-scale production of homogeneous organoids remains challenging, thereby limiting their applicability in high-throughput drug screening.

Tumor organoid culture protocols and conditions vary considerably among different tumor types [118]. Patient-derived GBM tissue collected from needle biopsies or surgical resections is widely used to establish multicellular GBM cultures in vitro under defined non-adherent conditions [119]. A recent study has advanced the generation of patient-derived GBM organoids (GBOs) by establishing a rapid and reliable culture system [120]. This platform enables the efficient production and biobanking of GBOs for downstream applications, including drug testing, and offers several advantages over conventional tumor organoid models. GBOs have been shown to recapitulate key features of their parental tumors, including histopathological characteristics, intratumoral heterogeneity, gene expression programs, and mutational profiles [120]. Following intracranial implantation into animal models, GBOs display rapid tumor formation and invasive growth, supporting their utility as a platform for in vivo studies [120]. GBOs can also be used to evaluate immunotherapeutic responses. For example, a phase I study investigating dual-targeting chimeric antigen receptor (CAR)-T cells (EGFR–IL13Rα2 CAR-T cells) in patients with recurrent glioblastoma demonstrated that GBOs enable real-time assessment of CAR-T cell bioactivity [121].

Collectively, tumor organoids and brain organoid-based models provide complementary 3D in vitro platforms for GBM research. GBOs preserve key histological, genetic, transcriptional, and cellular features of the parental tumors, including aspects of intratumoral heterogeneity and the TME [105]. In contrast, brain organoids enable the modeling of interactions between GBM cells and normal brain cell types, facilitating mechanistic investigation of tumor invasion, neural-tumor integration, and tumor-induced remodeling of brain-like tissue [112]. Combining these two model systems may therefore provide a more physiologically relevant platform for both fundamental and translational GBM research. Recent progress has led to the development of a complex chimeric culture system, termed individualized patient tumor organoids (IPTOs), in which cerebral organoids serve as host tissue to support the growth of resected patient tumor explants [122]. These chimeric organoids have been shown to preserve the cellular heterogeneity and molecular features of original tumors while providing a brain-like microenvironment for dissecting tumor-brain interactions [122]. IPTOs allow tumor-intrinsic programs and tumor-extrinsic brain microenvironmental cues to be investigated within the same platform, enabling the analysis of invasion, cellular plasticity, clonal selection, therapeutic response, and reciprocal interactions between malignant cells and surrounding neural tissue, thereby supporting their translational value as patient-specific platforms for functional drug testing and precision oncology.

### 3D bioprinted constructs

Regenerative engineering has spurred the development of state-of-the-art technologies such as 3D bioprinting to address the unmet need for allograft tissues and organs in clinical medicine. 3D bioprinting enables the in vitro fabrication of artificial multicellular tissues and organs that replicate their native hierarchical organization [123]. In general, 3D bioprinting precisely controls the spatial deposition of living cells and biocompatible materials, such as natural extracellular matrix components and synthetic polymers, layer by layer onto a substrate to create 3D constructs that resemble their biological counterparts [124]. The biomaterials used, comprising cells and diverse structural materials, are collectively termed ‘bioinks’ [125]. These bioinks are designed to provide the necessary mechanical support and biological cues that sustain cell viability, promote proliferation, and guide differentiation [125]. This technology has accelerated the development and application of various model systems, including scaffold-free and scaffold-based constructs, organ-on-a-chip platforms, cancer models, and digital light processing (DLP)-based 3D systems [126, 127]. 3D bioprinting has become a platform for the in vitro generation of functional tissues and organs, including the heart and liver, and for the large-scale fabrication of clinically relevant biomimetic tissues [126, 127]. The capacity of 3D bioprinting to spatially organize multiple cell types while precisely controlling microenvironmental viscosity and stiffness makes it a useful tool for investigating tumor biology. Accordingly, bioprinting-based cancer models have been developed for a variety of tumor types, including lung, skin, prostate, and brain cancers [128, 129].

**Fig. 4:**
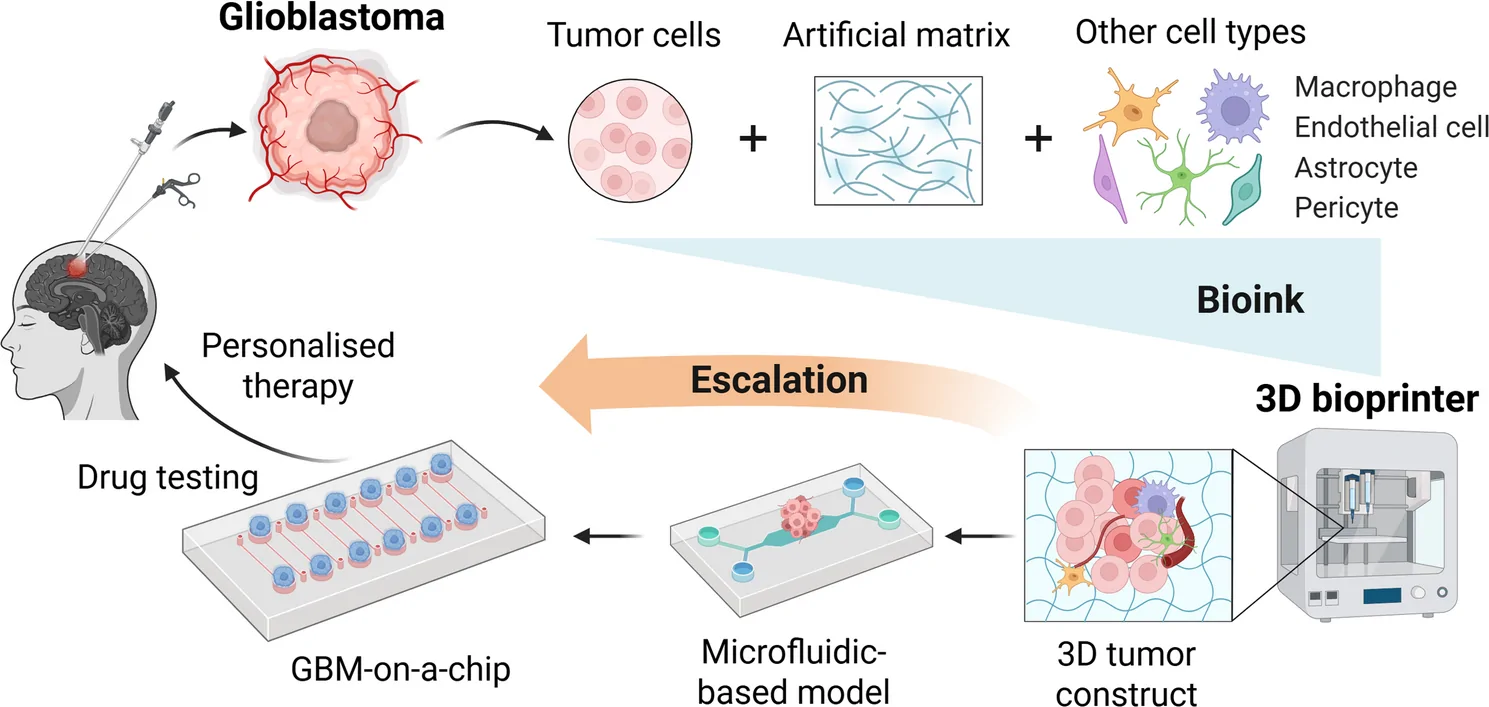
3D bioprinting-based GBM modeling and its application in personalized therapy. A strategy for modeling GBM using 3D bioprinting technologies. Tumor cells isolated from GBM tissue are combined with artificial extracellular matrices and various microenvironmental cell types, including macrophages, endothelial cells, astrocytes, and pericytes, to form a composite bioink. The bioink is then utilized by a 3D bioprinter to generate complex, multicellular 3D tumor constructs that mimic the native tumor architecture and cellular heterogeneity. 3D tumor constructs can be further integrated into microfluidic-based platforms, which simulate vascular flow and enhance the physiological relevance. With increasing system complexity, these models can be escalated into GBM-on-a-chip platforms, which enable high-throughput drug screening and functional testing under controlled microenvironmental conditions

The complexity of the GBM TME has attracted considerable attention relative to other tumor types. In recent years, researchers have increasingly sought to recapitulate this microenvironment using 3D bioprinting technologies (Fig. 4). Bioprinting may offer solutions to several limitations of organoid models, including batch-to-batch variability, prolonged cultivation times, and low production yields. Early studies developed relatively simple 3D bioprinting systems to investigate GSC growth, phenotypic transitions, vascular induction within artificial extracellular matrices, and therapeutic responses [130, 131]. These studies demonstrated that scaffold-based artificial matrices can provide a supportive microenvironment for GSC proliferation and differentiation [130, 131]. Moreover, compared with other modeling systems, bioprinting techniques offer distinct advantages for fabricating vascularized tumor models, as they enable the precise spatial positioning of endothelial cells within 3D constructs prior to the incorporation of additional cell types. Nonetheless, several factors may affect the investigation of microvascular function within the TME, including the biocompatibility and biodegradability of biomimetic materials used in vascular constructs, which can influence angiogenesis, vascular remodeling, and cellular responses. Furthermore, vascular networks in many bioprinted GBM models remain non-perfusable and therefore do not fully recapitulate authentic blood vessels. One study addressed this limitation by generating perfusable blood vessels within a 3D tumor model using a sacrificial bioink composed of a thermoreversible, biocompatible synthetic polymer to create an embedded vascular network [53]. Using this platform, the authors further investigated cell–cell interactions by incorporating a tumor-specific bioink containing GSCs, astrocytes, and microglia. These findings support the use of this 3D bioprinted model as a clinically relevant platform for mechanistic studies and drug screening [53].

3D bioprinting offers a unique approach for studying complex interactions between tumor and non-tumor cells by enabling the spatially controlled construction of multiscale architectures composed of diverse cell types [132, 133]. Bioprinted GBM models have shown that GSCs can recruit macrophages and promote their polarization toward tumor-associated macrophage-like phenotypes, which in turn enhance GBM growth and invasion [53, 132, 133]. These findings are consistent with in vivo evidence that GSC-derived factors, such as periostin, recruit M2-like tumor-associated macrophages and promote malignant progression [134]. In addition, patient-derived bioprinted GBM microenvironments incorporating GSCs, macrophages, astrocytes, and neural precursor cells have been shown to recapitulate clinically relevant transcriptional programs associated with stemness, invasion, immune interactions, and drug resistance [132, 133]. Because GBM evolution and heterogeneity are shaped by TME conditions, including hypoxia, inflammation, and angiogenesis, vascularized and perfusable bioprinted GBM platforms enable the investigation of TME-driven heterogeneity, immune modulation, vascular niche-dependent responses, and treatment-resistant tumor states.

Microfluidics, an emerging miniaturized technology for manipulating microscale samples, has been widely applied to develop high-throughput drug-screening systems in cancer research [135]. In GBM research, microfluidic platforms enable precise control of fluid flow, chemical gradients, shear stress, vascular architecture, and tumor–stromal organization within defined 3D microenvironments [136]. A study established a 3D microfluidic-based vascularized GBM model that replicates blood vessel architecture through the self-assembly of endothelial cells, astrocytes, and pericytes [137]. This system has proven useful for assessing the efficacy of GBM-targeted nanoparticles in crossing the blood-brain barrier in vitro [137, 138]. In addition, organ-on-a-chip (OoC) technology, which emulates organ- or tissue-level functions on microfabricated devices in vitro, offers a platform for accelerating drug screening pipelines [139]. By integrating microfluidics, organ-on-a-chip technology, and 3D bioprinting, researchers have developed GBM-on-a-chip platforms for evaluating patient-specific therapeutic responses under diverse treatment conditions [140–142]. In this system, microfluidic approaches establish radial gradients of oxygen and metabolites that mimic key biochemical features of the TME, including the perivascular niche characteristic of GBM [140]. Additionally, this platform enables evaluation of drug combinations and assessment of their potential toxicity to normal brain tissue [141, 142]. Collectively, GBM-on-a-chip models provide platforms for mechanistic studies of microenvironment-driven heterogeneity, blood-brain barrier-associated drug delivery, therapeutic resistance, and personalized treatment selection. However, several challenges remain, including the limited availability of bioinks capable of recapitulating the extensive heterogeneity of GBM, concerns regarding the biocompatibility and biodegradability of biomimetic materials, and the need for further advancements in printing modalities.

## Organotypic brain slice cultures

**Fig. 5:**
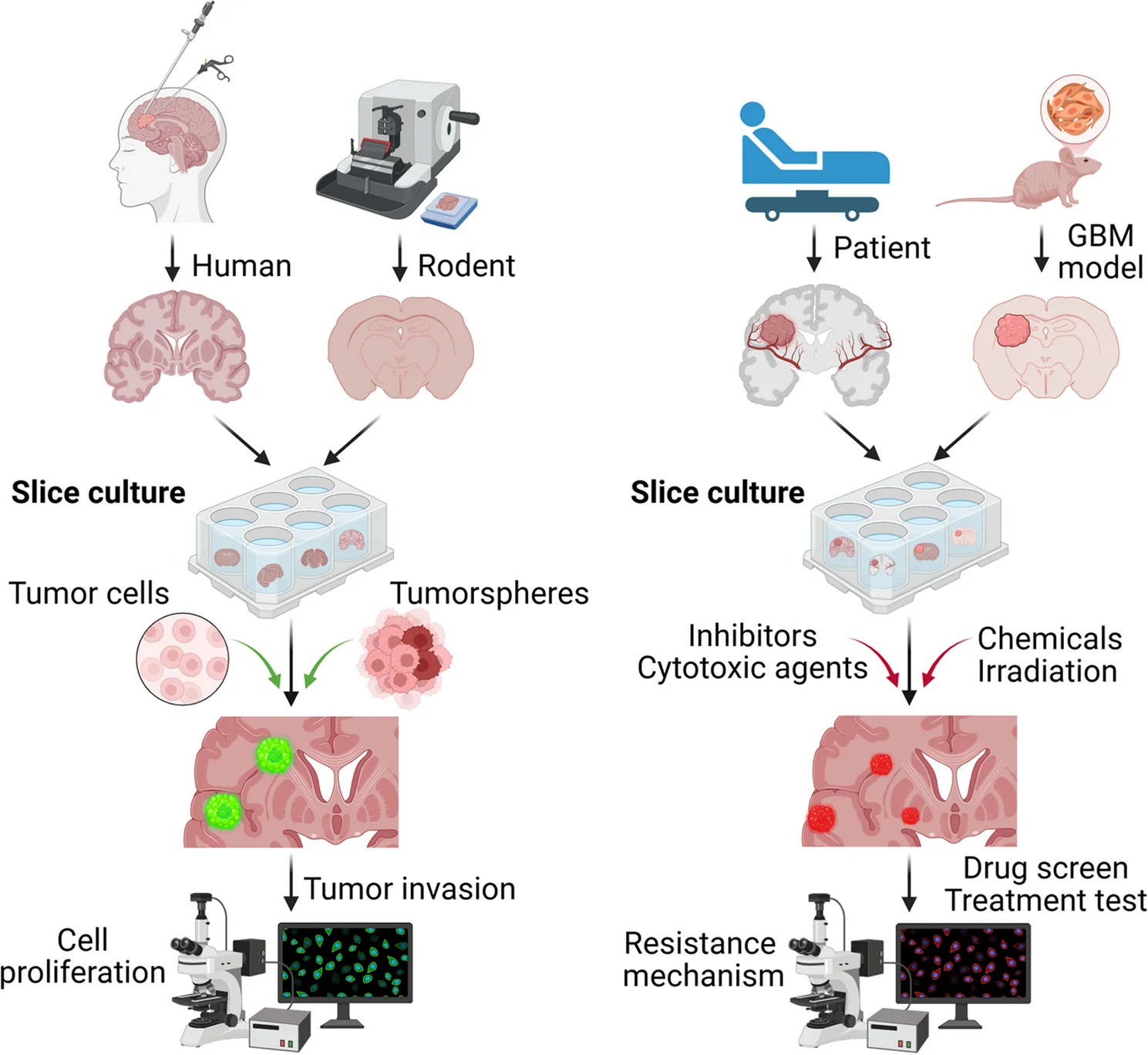
Organotypic brain slices for GBM study. Schematic representation of human- and rodent-derived organotypic brain slice culture systems used to investigate GBM biology and therapy. Ex vivo brain slices prepared from surgical or experimental specimens are maintained under controlled culture conditions. These systems enable the assessment of tumour invasion, proliferation, drug efficacy, cytotoxicity, and resistance mechanisms following treatment with inhibitors, chemotherapeutic agents, or irradiation, providing a physiologically relevant platform for mechanistic studies and preclinical testing

The organotypic brain slice culture model was first developed in the 1960s and has since been widely used by neuroscientists to study neurophysiological processes [143]. In 1998, it was first reported as a method for investigating the invasive properties of brain tumors [144], and it has subsequently been widely used to dissect the mechanisms underlying GBM invasion [145]. Today, this model is well established for studying neuronal function and neurological disorders such as neurodegeneration and brain tumors. Broadly, two main approaches are used to generate GBM brain slice culture models of GBM (Fig. 5). One method involves using brain tissue obtained directly from surgical specimens from GBM patients or tumor-bearing animals. Alternatively, normal rodent or human brain tissue, typically obtained during neurological surgery, can be co-cultured with GBM cells. Ex vivo brain slices are often prepared via a vibratome that can generate sections ranging from 200 to 400 μm in thickness. Under specialized culture media and controlled conditions, these slices can be maintained for several weeks, enabling long-term ex vivo studies [146]. However, tissue slicing protocols and culture conditions vary considerably across laboratories worldwide [147]. Depending on specific biological objectives, research groups have adopted distinct variations of ex vivo brain slice models, along with different imaging methods such as endpoint fluorescence imaging and time-lapse confocal microscopy [148]. For example, some models involve implantation of GSCs into cultured brain slices to investigate tumor cell proliferation and migration within the native brain microenvironment, whereas others utilize direct seeding of tumorspheres onto brain slices to study tumor invasion. Compared with in vitro models such as brain organoids, organotypic brain slices preserve the native cellular architecture and cellular diversity of neural tissue, providing a physiologically relevant platform for studying GBM–microenvironment interactions. These models are particularly useful for assessing GSC invasiveness, malignant behavior, and responses to candidate therapies targeting GBM progression.

Over the past two decades, this model system has undergone substantial development and has been increasingly applied in GBM research. Studies using rodent-derived brain slices have shown that GBM invasion patterns in these models closely resemble those observed in vivo [146, 149, 150]. Mouse-derived model systems have progressed from using postnatal brain tissue cultured in serum-containing media to using adult brain slices maintained under serum-free conditions for several weeks [146]. Patient-derived GSCs have been shown to exhibit regional preferences, with the subependymal area providing a permissive microenvironment for their survival when engrafted into adult mouse brain slices from different anatomical regions [146]. Many research groups have focused on establishing organotypic brain slice cultures from human brain tissue [146–148]. One study introduced patient-derived primary GBM cells into ex vivo–cultured human brain slices prepared from freshly resected neocortical tissue obtained from patients undergoing epilepsy surgery [147]. This platform recapitulated key features of GBM biology, including tumor growth and infiltration dynamics, responsiveness to temozolomide, and cytokine expression profiles. It also enabled the live isolation and analysis of specific cell types, such as astrocytes and neurons, to assess local microenvironmental changes during GBM progression. In another study, non-tumor brain samples were collected from patients undergoing corticectomies or from tissue within the planned supramarginal resection zone [145]. Primary human GBM *IDH*-wildtype cells, labeled with green fluorescent protein (GFP), were then cultured on organotypic brain slices [145]. The tumor cells preferentially migrated along perivascular spaces, while their invasive behavior was modulated by interactions with vascular structures and exposure to hypoxic conditions [145]. Additional studies using this model have shown that sialic acid turnover is required for GBM growth and tumor cell network formation, and that CD44 expression in myeloid cells contributes to the regulation of GBM invasiveness [151, 152].

Increasing efforts have focused on generating slice cultures directly from tumor tissue to enable real-time monitoring of tumor cell invasion and assessment of therapeutic sensitivity. GBM slice cultures provide a patient-relevant platform for assessing interpatient variability in tumor cell migration and responses to anti-migratory therapies. For example, *EGFR*-amplified GBMs exhibit increased migratory capacity, and treatment with the EGFR inhibitor gefitinib selectively reduced tumor cell migration in *EGFR*-amplified tumors, suggesting that EGFR inhibition may represent a genotype-informed anti-invasive strategy for selected patients [153, 154]. The interferon regulatory factor 3 (IRF3) pathway has also been shown to negatively regulate GBM invasion in ex vivo models by suppressing the expression of extracellular matrix-associated gene networks [155]. Furthermore, GBM slice cultures have been used to evaluate responses to irradiation, heavy-ion exposure, and temozolomide, as well as to investigate mechanisms of therapeutic resistance [156]. These slices preserve key histopathological features of individual tumors and have revealed marked interpatient differences in treatment sensitivity, supporting their value for patient-specific ex vivo therapeutic testing [154, 156]. However, their broader application is limited by the availability and quality of fresh tumor tissue, short-term culture viability, protocol variability, and limited capacity to model processes such as angiogenesis, systemic immune responses, and tumor evolution over extended time scales.

## In vivo modeling of GBM

**Fig. 6:**
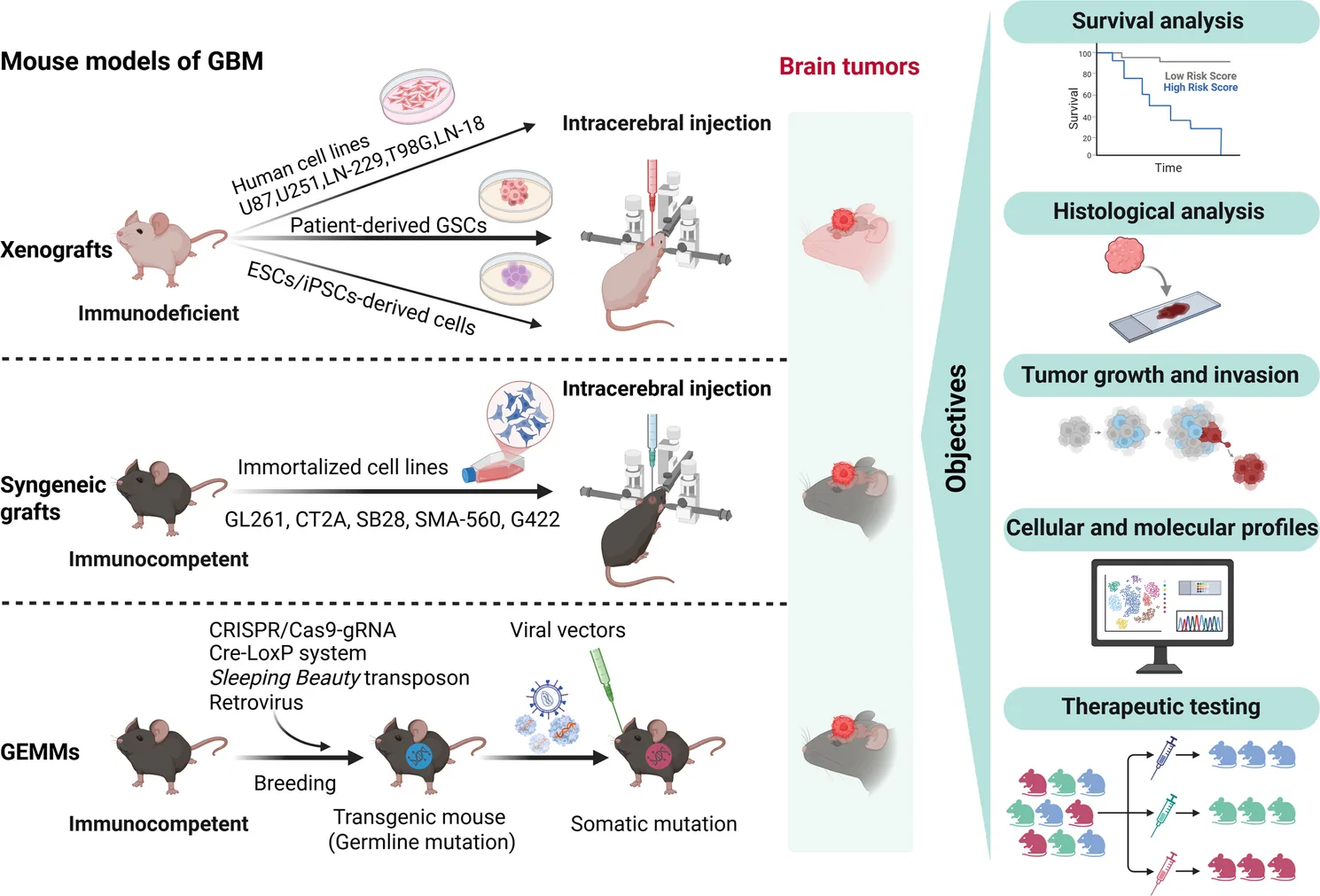
In vivo modeling and its applications in preclinical research. Three major mouse model systems are used to study GBM: xenografts, syngeneic grafts, and genetically engineered mouse models (GEMMs). (1) In xenograft models, human-derived GBM cells (including established cell lines such as U87, U251, LN-229, T98G, and LN-18, patient-derived GSCs, or ESC/iPSC-derived malignant cells) are injected intracerebrally into immunodeficient mice to study GBM biology in vivo. (2) Syngeneic graft models utilize murine GBM cell lines (e.g., GL261, CT2A, SB28, SMA-560, G422) injected into the brains of immunocompetent mice, enabling investigation of tumor-immune interactions in an intact immune system. (3) GEMMs are developed in immunocompetent mice through both germline and somatic genetic engineering using CRISPR/Cas9, Cre-*loxP*, *Sleeping Beauty* transposons, or viral vectors, allowing for spontaneous tumor formation in a genetically defined context. These models serve multiple research objectives, including survival analysis, histological characterization, assessment of tumor growth and invasion, cellular and molecular profiling, and therapeutic testing, providing complementary platforms for understanding GBM pathobiology and advancing preclinical drug development

Mouse models are among the most widely utilized in vivo experimental systems in biomedical research. In cancer studies, they play key roles in elucidating tumor biology and progression, as well as in evaluating the safety and efficacy of therapeutic interventions [157]. Mouse models of GBM (Fig. 6) are widely used to model the TME, validate in vitro and ex vivo findings, and evaluate potential therapeutic agents, although inherent cross-species differences may affect their clinical translatability [158]. The advantages of these models include genetic and physiological similarities to humans, low inter-individual variability, ease of genetic manipulation, high reproducibility and scalability in preclinical settings, and suitability for investigating the multifaceted mechanisms underlying tumor‒host interactions. Limitations such as the high cost and time required to generate and maintain specific mouse strains are common across many areas of scientific research. Nevertheless, compared with other fields, therapies that demonstrate efficacy in GBM mouse models are disproportionately more likely to fail in human clinical trials, underscoring the need for improved model design and greater translational relevance [158]. In general, in vivo GBM models can be broadly classified into three categories based on the origin of tumor cells and the immune status of the host: xenograft models, syngeneic models, and genetically engineered mouse models (GEMMs).

### Xenografts and syngeneic grafts

Both xenograft and syngeneic models involve the orthotopic implantation of tumor cells into the mouse brain to induce GBM formation. Xenograft models typically entail the direct implantation of human GBM cells, including established cell lines, genetically modified NSCs, or patient-derived GSCs, into the brains of immunodeficient or humanized mice, such as nude, NOD-SCID, and NSG strains [159, 160]. In contrast, syngeneic models involve the injection of murine GBM cells into immunocompetent mice, such as the C57BL/6 strain, which retain intact immune systems [64]. Therapeutic responses can be rapidly measured using these model systems, which require relatively less time and lower cost than GEMMs, the latter of which usually demand considerable effort to intercross mouse lines with defined genetic backgrounds [161].

#### Patient-derived xenografts

Xenograft models aim to recapitulate human GBM through the propagation of human tumor cells in mice. Common approaches include cell line-based xenografts and patient-derived xenografts (PDXs). Established human glioma cell lines, including U87, U251, T98G, LN-18, and LN-229, have been widely used to generate xenografts [162]. These cell lines can be propagated and maintained in serum-containing media; however, prolonged passaging may lead to genetic drift and increase the risk of cross-contamination [163]. In addition, the extent to which these models faithfully recapitulate the biological characteristics of human GBM remains questionable, given the well-documented intra- and intertumoral heterogeneity of the disease [160, 161]. Human GBM cell lines derived from NSCs or iPSCs have also been utilized to establish xenograft models, although stem cell-based approaches remain associated with ethical considerations. Currently, cell line-based xenografts are commonly applied to studies of tumor-intrinsic signaling network and angiogenesis, whereas increasing evidence supports the use of PDXs as a more clinically relevant alternative [164].

PDX models are generated using GBM tissue collected from patients undergoing surgical resection. Fresh tumor fragments, dissociated single-cell suspensions, or minimally passaged patient-derived GSCs are implanted into the brains of recipient mice to preserve patient-specific tumor features as closely as possible [164]. These PDXs better mimic the key characteristics of human GBM than other known model systems, including diffuse invasion, angiogenesis, pseudopalisading necrosis, cellular heterogeneity, and patient-specific molecular alterations [160, 165]. In some models, invasive dissemination of human GBM cells through the corpus callosum into the contralateral hemisphere has been observed [163]. In addition, PDX models enable the identification of distinct biological features associated with different GBM subtypes, including classical, proneural, and mesenchymal, which are defined by tumor cell transcriptional profiles [166, 167]. For example, PDXs established from mesenchymal-subtype GBMs exhibit greater invasive capacity and increased angiogenic activity than those derived from the proneural subtype [167]. In addition, PDX models have been shown to preserve the heterogeneous subclonal architecture present within individual GBM tumors [164]. Longitudinal PDX recurrence models have shown that chemoradiotherapy enriches pre-existing therapy-resistant mesenchymal-like and stem-like populations, including spatially distinct THY1-positive cells, supporting the use of these models to study recurrence-associated tumor evolution [168].

PDXs are widely used to evaluate patient-specific drug responses because they retain many of the histopathological and molecular features of the original tumors. However, variability among PDX lines and the limited availability of patient-derived material can compromise reproducibility and standardization across studies. More broadly, xenograft models provide opportunities to investigate key mechanisms associated with the hallmarks of human GBM, but their dependence on immunodeficient hosts limits their ability to model intact tumor–immune interactions, define the roles of specific immune components in tumor evolution, or evaluate immunotherapies. Moreover, conventional human GBM PDXs progressively acquire murine stromal components, further limiting their capacity to recapitulate fully human immune and stromal interactions. Therefore, xenograft models should be complemented by immunocompetent, humanized, organoid, and ex vivo systems when studying tumor–immune crosstalk [165].

#### Syngeneic grafts

Humanized mouse models reconstituted with defined human immune cell populations allow for more targeted investigation of immunomodulatory effects on GBM growth in vivo. Syngeneic models, generated by implanting murine GBM cells into immunocompetent hosts, are well suited for characterizing the tumor immune landscape and identifying potential immunotherapeutic strategies. Commonly used syngeneic tumor lines are derived from spontaneous murine tumors [169] or tumors induced by mutagenic chemicals or transposons, including SMA-560 [169, 170], GL261, CT-2 A, G422 [171], and SB28 [62]. These cell lines can be readily maintained and expanded in vitro prior to implantation into mice, reproducible tumor formation with relatively stable growth kinetics and predictable effects on host survival. Among them, GL261 and CT-2 A are the most extensively used in GBM research, whereas SB28 has been reported to more accurately recapitulate the mutational load and immune microenvironment of human GBM [62–64, 172]. Each syngeneic model exhibits distinct molecular, immunological, and pathological features suited to specific experimental applications. However, no single mouse model fully recapitulates the mechanistic complexity of human GBM, including intratumoral heterogeneity, stem-like populations, immune suppression, microenvironmental interactions, and responses to standard-of-care treatment, which partly contributes to the translational gap in GBM therapy, particularly immunotherapy [173]. Several representative model cell lines are introduced below.

##### GL261

GL261 cells are capable of forming tumorspheres and expressing stem cell markers, including CD133 and Nestin, when cultured in serum-free medium [174]. Tumors generated in GL261-bearing C57BL/6 mice exhibit histological features similar to those of human GBM. Depending on the implanted cell number and experimental design, GL261 tumors typically reach experimental endpoints within approximately 3–6 weeks following orthotopic implantation [175]. Standard treatments, including chemotherapy and radiotherapy, moderately extend survival in this model, partially mirroring therapeutic outcomes in human GBM [37, 64]. However, GL261 tumors exhibit higher immunogenicity and greater mutational burden than human GBM, generating more neoepitopes capable of eliciting antitumor immune responses [176, 177]. Additionally, GL261 cells display high MHC I expression, facilitating the presentation of these neoepitopes to immune cells [178]. This enhanced immunogenicity may lead to overestimation of immunotherapy efficacy in preclinical studies, limiting the direct translational relevance of GL261 models. For example, unlike the limited efficacy observed in human GBM clinical trials, GL261 tumors are responsive to checkpoint inhibitors targeting PD-1, CTLA-4, and IDO [179, 180]. In one study, GL261-bearing mice treated with combined checkpoint inhibition showed long-term survival and markedly reduced regulatory T cell (Treg) infiltration within the TME, an outcome rarely observed in human GBM [181]. Overall, the GL261 model may be well suited for investigating therapeutic approaches, including gene therapy, nanoparticle-based treatments, and treatment-associated changes within the brain tumor microenvironment.

##### CT-2 A

The CT-2 A line can also grow as tumorspheres under serum-free conditions. Compared with GL261, it is generally more tumorigenic and invasive, with C57BL/6 host mice often exhibiting shorter survival under the same implantation protocol [181]. Following intracranial implantation of 1 ⅹ 10⁴ CT-2 A cells, reported median survival is approximately 20 days, although survival varies with cell number, implantation site, and experimental design [165]. Although the CT-2 A model is less commonly used, it more accurately recapitulates several histopathological features of human GBM, including abundant vascularization, high cellular density, increased proliferative activity, and pseudopalisading necrosis [182]. Genetically, CT-2 A tumors retain wild-type *p53* but lack the tumor suppressor *PTEN*, a profile that parallels certain human GBM subtypes [182–184]. Furthermore, unlike GL261, CT-2 A tumors exhibit distinct mutational and transcriptional profiles that contribute to differential responses to both conventional treatments and immunotherapies. Compared with GL261, CT-2 A tumors appear more sensitive to radiation but less responsive to temozolomide treatment [185, 186]. However, these findings require further validation, as CT-2 A remains less extensively characterized. Current studies using CT-2 A models are largely focused on exploring potential immunotherapeutic strategies for human GBM. CT-2 A tumors express low levels of MHC I and exhibit defects in intrinsic antigen presentation machinery [187]. The TME of CT-2 A tumors is enriched in dysfunctional CD4^+^ T cells, which are associated with CD8^+^ T cell exhaustion and a lack of responsiveness to PD-1 blockade [188]. Accordingly, CT-2 A models exhibit lower immunogenicity than GL261 models while more closely resembling the immunosuppressive features of human GBM. In addition, CT-2 A cells are resistant to IFNγ-induced cell death, a phenotype linked to defects in intracellular interferon signaling pathways [189]. CT-2 A tumors are highly aggressive in vivo, exhibiting rapid proliferation and a mesenchymal-like phenotype [189]. Although certain features of the CT-2 A model remain incompletely characterized, this model is expected to be useful for future studies evaluating immunotherapeutic candidates for GBM, including antigen-directed immunotoxins.

##### SB28

SB28 cells can be readily expanded in vitro, and implantation of a small number of cells into immunocompetent C57BL/6 mice is sufficient to induce orthotopic tumor formation [37, 62]. Following intracranial implantation, SB28-bearing mice typically reach experimental endpoints within approximately 19–23 days, depending on the implanted cell number and study design [165, 190]. Although relatively new compared with other syngeneic models, SB28 closely recapitulates several key features of human GBM, including histopathology, mutational burden, intratumoral heterogeneity, low immunogenicity, and an immunosuppressive TME [37, 62]. SB28 tumors are resistant to radiotherapy, temozolomide, and immune checkpoint blockade, reflecting the therapeutic refractoriness of human GBM [63, 64, 172]. Consistent with human GBM, the SB28 TME contains few functional CD8^+^ T cells and abundant immunosuppressive myeloid populations, including microglia and macrophages/monocytes [37, 63]. Recent studies have further used SB28 to evaluate therapeutic strategies designed to overcome immune-cold and therapy-resistant GBM phenotypes, including combination immunotherapy and oncolytic virotherapy approaches [191]. Thus, SB28 provides a platform for preclinical therapeutic development, particularly in immunotherapy research, although its applicability to personalized GBM modeling remains limited.

##### Other lines

In recent years, other syngeneic glioma lines, such as SMA-560 [170] and G422 [192], have been used less frequently in GBM studies. The SMA-560 model, established in VM/Dk mice, remains less extensively characterized than GL261, CT-2 A, and SB28 but may be useful for investigating TGF-β-driven immunosuppression and invasion, as SMA-560 cells produce high levels of TGF-β [193, 194]. Histologically, SMA-560 tumors resemble anaplastic astrocytoma rather than GBM [169, 193], which may restrict their applicability in GBM therapeutic development. The G422 line was originally derived from gliomas established in KM mice and has mainly been used to investigate antitumor immunotherapies, vaccine-based approaches, drug delivery strategies, and locoregional treatment platforms [192, 195]. In addition, syngeneic models can be generated using genetically modified mouse NSCs, such as those carrying *Pten* deletion [196]. Overall, although these less commonly used syngeneic models have specific limitations, they provide additional platforms for investigating the immune landscape, tumor evolution, and mechanisms of therapeutic resistance in GBM.

### Engineered mouse models

GEMMs represent an important in vivo system for modeling the spontaneous initiation and progression of human GBM [161]. They are typically generated using genetic engineering approaches such as viral transduction, transposon-mediated gene delivery, Cre-*loxP* recombination, and CRISPR/Cas9-mediated genome editing [161, 197]. Because TICs arise from endogenous genetic alterations and expand within an intact brain microenvironment, GEMMs are particularly useful for studying gliomagenesis, subclonal evolution, tumor–host interactions, invasion, angiogenesis, and immunophenotypic changes. Compared with transplantation-based models, GEMMs are more suitable for investigating how defined genetic alterations shape GBM heterogeneity and evolution [198], as well as for evaluating therapeutic strategies, including nanodrugs, nucleotide-based agents, and CAR immune cell therapies [199–202]. However, GEMMs remain limited by high cost, long breeding timelines, variable tumor latency, and incomplete representation of patient-specific intratumoral heterogeneity.

Several key genetic and epigenetic alterations implicated in driving gliomagenesis have been strategically manipulated to generate GEMMs for GBM modeling [158]. These include loss-of-function alterations in tumor suppressor genes such as *Pten*, *p53*, *Ink4a*/*Arf*, *Nf1*, *Cdkn2a*/*2b*, and *Rb*, as well as aberrant activation of oncogenic pathways involving PDGFR, EGFR, PI3K, KRAS, CDK4, and MDM2 [158]. Viral transduction is one of the most widely used approaches for introducing genetic modifications into target cells, with adenovirus, adeno-associated virus, lentivirus, and retrovirus commonly delivered intraventricularly or intracranially using stereotaxic equipment [203, 204]. Among these approaches, the RCAS–TVA retroviral vector system has shown substantial utility in preclinical glioma modeling [205, 206]. Transposon-based gene manipulation has also gained increasing attention in cancer research. Transposons are mobile genetic elements capable of relocating DNA sequences within the genome, among which *Sleeping Beauty* (SB) and *piggyBac* (PB) are the most widely used eukaryotic DNA transposons. Several studies have employed SB-based vector systems to efficiently generate GBM models [207–209], although the development of transposon-based models often requires 6 to 8 months. The Cre-*loxP* recombination system, which enables temporally and spatially controlled site-specific recombination, has also been widely used to generate transgenic mouse models [210]. Mice carrying gene sequences flanked by *loxP* sites are frequently crossed with mice expressing Cre recombinase under the control of cell type- or lineage-specific promoters, thereby generating conditional knockout or overexpression models. In GBM research, *Nestin* or *GFAP* promoters are commonly used to drive Cre expression in NSCs or glial cells [161, 211]. However, generating these models typically requires more than one year. To accelerate model development, viral vectors encoding Cre recombinase are increasingly used as an alternative approach. CRISPR/Cas9 is also highly effective for generating GEMMs carrying single or multiple gene mutations [206, 212]. Compared with conventional approaches, CRISPR/Cas9 enables more rapid model establishment and supports in vivo screening of tumor suppressor genes implicated in GBM pathogenesis. The major limitations of CRISPR/Cas9-based approaches include dependence on specific protospacer adjacent motif (PAM) sequences for single-guide RNA (sgRNA) recognition and the risk of off-target effects [213]. Two or more of these approaches can be combined to generate GEMMs, thereby expanding the diversity of preclinical GBM models. Recent studies have combined RCAS/tv-a systems with CRISPR/Cas9 to generate genetically defined primary glioma models, increasing the flexibility of somatic GEMM generation [214]. Together, these models are likely to improve our understanding of the GBM TME and its evolutionary trajectories and aid in the identification of novel therapeutic targets.

## Multi-omics and AI-assisted modeling of GBM

GBM evolution is driven by coordinated changes across genetic, epigenetic, transcriptional, spatial, and microenvironmental layers. Integrating multi-omics data from these layers with AI-assisted computational modeling can help elucidate how tumor cells acquire, maintain, or transition between malignant states during disease progression and therapeutic response [5, 23]. The integration of single-cell transcriptomic and epigenomic data with spatial transcriptomics is particularly useful because it links malignant cellular states, regulatory programs, chromatin accessibility, and immune or stromal populations to defined anatomical niches, such as hypoxic regions, invasive margins, vascular niches, and immune-enriched areas [8, 215, 216]. Spatially resolved multi-omics studies have shown that GBM cellular states are spatially organized and shaped by inflammatory, metabolic, and tumor-host interactions, while longitudinal single-cell multi-omics analyses of paired primary and recurrent GBM have revealed a therapy-associated shift toward mesenchymal states regulated in part by AP-1 activity [8, 215].

AI-assisted computational modeling can further improve tumor evolution inference by extracting informative patterns from high-dimensional multi-omics, imaging, and clinical datasets [217, 218]. Machine learning and deep learning approaches can integrate genomic alterations, transcriptomic states, epigenetic programs, radiomic features, histopathological images, treatment history, and clinical outcomes to predict molecular subtype, recurrence risk, survival, and therapeutic response. Recent reviews have emphasized the potential of AI-driven multi-omics approaches in GBM for molecular profiling, prognosis prediction, treatment stratification, and personalized medicine [219]. In parallel, AI-guided integration of computational modeling, intelligent materials, and advanced imaging has been proposed to support patient-specific and scalable neuro-oncology nanomedicine [220]. When combined with mechanistic constraints, AI models may help identify evolutionary trajectories, infer cell-state transitions, prioritize regulatory nodes, and predict which tumor subpopulations are likely to persist under specific therapeutic pressures.

Purely descriptive omics analyses can identify tumor states or patient subgroups but often fail to explain causality, temporal order, clonal fitness, or therapeutic selection. Mechanistic modeling can address these gaps by incorporating biological principles such as mutation accumulation, clonal competition, cellular plasticity, ligand-receptor signaling, metabolic constraints, and therapy-induced selection. AI-assisted approaches enhance pattern recognition and predictive capacity, and mechanistic modeling adds causal structure and interpretability. Together, these integrative approaches can improve reconstruction of GBM evolutionary trajectories, prediction of treatment response, patient-specific risk stratification, rational model selection, and identification of combination therapies targeting both tumor-intrinsic programs and tumor-extrinsic microenvironmental dependencies.

## Conclusions

Over the past decade, advances in single-cell profiling, spatial transcriptomics, lineage tracing, organoid culture, 3D bioprinting, genetically engineered models, and computational modeling have substantially improved our understanding of GBM evolution and therapeutic resistance. GBM remains largely incurable because malignant cells continuously adapt to selective pressures imposed by the brain microenvironment and therapy. This adaptive capacity is reflected in intratumoral heterogeneity, diffuse invasion, immunosuppressive myeloid-rich niches, therapy-resistant cellular states, and the molecular divergence between primary and recurrent tumors.

A key conceptual challenge is to distinguish hierarchical cancer stem cell models from dynamic state plasticity models. In the classical hierarchical model, GSCs are viewed as a relatively stable tumor-propagating subpopulation with self-renewal capacity. In contrast, recent single-cell and lineage-tracing studies support a more plastic view, in which stem-like properties can be reversibly acquired or lost as GBM cells transition among proneural-like, classical-like, mesenchymal-like, invasive, and therapy-tolerant states. These models are not mutually exclusive. Hierarchical organization may dominate in certain genetic or experimental contexts, whereas dynamic plasticity may become more evident under hypoxia, inflammatory signaling, immune selection, or therapeutic pressure. This distinction is particularly relevant to proneural-to-mesenchymal transition, which can be promoted by treatment and microenvironmental stress and is associated with recurrence, invasion, immune remodeling, and resistance.

Current GBM models each capture only selected aspects of this complexity. Patient-derived GSCs and organoids preserve tumor-intrinsic heterogeneity and support individualized drug testing, but they incompletely reproduce immune, vascular, and neural interactions. Scaffold-based, hydrogel, and 3D-bioprinted systems enable controlled interrogation of extracellular matrix cues, macrophage interactions, vascular niches, and drug delivery, but they still require improved standardization and scalability. Syngeneic and engineered mouse models provide immune-competent contexts for studying tumor evolution and immunotherapy, yet no single model fully recapitulates human GBM heterogeneity, recurrence, and treatment history. Therefore, future studies should select models according to clearly defined mechanistic questions rather than relying on any single platform as a universal representation of GBM.

Several key questions should guide future model development. First, how do specific genetic alterations, epigenetic programs, and microenvironmental signals jointly regulate transitions between stem-like, mesenchymal, invasive, and therapy-tolerant states? Second, which model systems best capture clonal selection and recurrence after surgery, radiotherapy, temozolomide, targeted therapy, or immunotherapy? Third, how can immune, vascular, neural, and metabolic components be incorporated into patient-specific platforms without sacrificing reproducibility, throughput, and interpretability? Fourth, how can multi-omics and AI-assisted computational modeling be embedded into experimental systems to infer tumor evolution and predict treatment response with mechanistic accuracy?

Future progress will require integrated model pipelines rather than isolated systems. Patient-derived organoids, bioprinted vascularized platforms, immune-competent models, longitudinal single-cell and spatial multi-omics, and AI-assisted mechanistic modeling should be combined to reconstruct tumor evolution across spatial, temporal, and therapeutic dimensions. Finally, personalized GBM modeling must address practical and regulatory barriers before broad clinical implementation. These include ethical governance of patient-derived tissues and genomic data, informed consent for biobanking and AI-based prediction, reproducibility across laboratories, cost and scalability of organoid or bioprinted platforms, quality control standards, clinically compatible turnaround times, and regulatory validation of model-guided therapeutic decisions. Addressing these issues will be essential for translating advanced GBM models from exploratory research tools into reliable platforms for precision neuro-oncology.

## Data Availability

No datasets were generated or analysed during the current study.

## Abbreviations

AI: artificial intelligence
AC-like: astrocyte-like
CRISPR: clustered regularly interspaced short palindromic repeats
DLP: digital light processing
EBs: embryoid bodies
EGF: epidermal growth factor
ESCs: embryonic stem cells
FGF-2: fibroblast growth factor 2
GBM: glioblastoma
GBOs: glioblastoma organoids
GEMMs: genetically engineered mouse models
GFP: green fluorescent protein
GLICO: glioma cerebral organoids
GSCs: glioblastoma stem cells
ILK: integrin-linked kinase
iPSC: induced pluripotent stem cells
IPTO: individualized patient tumor organoid
IRF3: interferon regulatory factor 3
MES-like: mesenchymal-like
NMU: nitrosomethylurea
NPC-like: neural progenitor-like
NSCs: neural stem cells
OoC: organ-on-a-chip
OPC-like: oligodendrocyte progenitor-like
PAM: protospacer adjacent motif
PB: piggyBac
PCL: polycaprolactone
PDXs: patient-derived xenografts
PEG: polyethylene glycol
PGA: polyglycolic acid
PLA: polylactic acid
PMT: proneural-to-mesenchymal transition
RCAS: replication-competent ASLV long terminal repeat (LTR) with a Splice acceptor
SB: Sleeping Beauty
sgRNA: single guide RNA
SGZ: subgranular zone
TAMs: Tumor-associated macrophages
TICs: tumor-initiating cells
TME: tumor microenvironment
Treg: regulatory T cell
WHO: World Health Organization
2D: two-dimensional
3D: three-dimensional
